# Nitric Oxide Synthase-3 Promotes Embryonic Development of Atrioventricular Valves

**DOI:** 10.1371/journal.pone.0077611

**Published:** 2013-10-29

**Authors:** Yin Liu, Xiangru Lu, Fu-Li Xiang, Man Lu, Qingping Feng

**Affiliations:** 1 Department of Physiology and Pharmacology, University of Western Ontario, London, Ontario, Canada; 2 Department of Medicine, University of Western Ontario, London, Ontario, Canada; 3 Lawson Health Research Institute, London, Ontario, Canada; 4 Department of Ultrasound, Sichuan Academy of Medical Sciences, Sichuan Province People’s Hospital, Chengdu, Sichuan, China; Leiden University Medical Center, The Netherlands

## Abstract

Nitric oxide synthase-3 (NOS3) has recently been shown to promote endothelial-to-mesenchymal transition (EndMT) in the developing atrioventricular (AV) canal. The present study was aimed to investigate the role of NOS3 in embryonic development of AV valves. We hypothesized that NOS3 promotes embryonic development of AV valves via EndMT. To test this hypothesis, morphological and functional analysis of AV valves were performed in wild-type (WT) and NOS3^−/−^ mice at postnatal day 0. Our data show that the overall size and length of mitral and tricuspid valves were decreased in NOS3^−/−^ compared with WT mice. Echocardiographic assessment showed significant regurgitation of mitral and tricuspid valves during systole in NOS3^−/−^ mice. These phenotypes were all rescued by cardiac specific NOS3 overexpression. To assess EndMT, immunostaining of Snail1 was performed in the embryonic heart. Both total mesenchymal and Snail1^+^ cells in the AV cushion were decreased in NOS3^−/−^ compared with WT mice at E10.5 and E12.5, which was completely restored by cardiac specific NOS3 overexpression. In cultured embryonic hearts, NOS3 promoted transforming growth factor (TGFβ), bone morphogenetic protein (BMP2) and Snail1expression through cGMP. Furthermore, mesenchymal cell formation and migration from cultured AV cushion explants were decreased in the NOS3^−/−^ compared with WT mice. We conclude that NOS3 promotes AV valve formation during embryonic heart development and deficiency in NOS3 results in AV valve insufficiency.

## Introduction

Valvular heart disease is a significant health problem and contributes to more than 44,000 deaths in United States annually [1]. The causes of valvular heart disease include congenital malformation and rheumatic disease which is secondary to bacterial infection. Congenital valve disease affects about 2% of the general population [2], [3]. The disease may manifest as valvular stenosis, an obstruction of outflow, or as regurgitation, a defective closure resulting in backward flow. Congenital valve malformations tend to cluster in families among both close and distant relatives, suggesting a genetic component of this disease [4], [5]. However, molecular mechanisms responsible for congenital valve disease are still not fully understood.

Nitric oxide synthase (NOS) enzymes convert L-arginine to L-citrulline and produce nitric oxide (NO), a signaling molecule involved in a wide range of physiological processes including apoptosis, angiogenesis, cell proliferation and differentiation [6]–[8]. Three distinct isoforms of NOS has been identified: neuronal NOS (NOS1), inducible NOS (NOS2) and endothelial NOS (NOS3). The expression of NOS3 starts around E9.5 during early mouse embryonic heart development [9]. This expression remains high until E13.5. By E14.5, the levels of NOS3 expression decrease in both atria and ventricles. After E19.5 low NOS3 levels are still detectable and this level of NOS3 expression in the myocardium remains into adulthood [9].

The atrioventricular (AV) valve development starts at E9 with the formation of endocardial cushions. A subpopulation of endocardial cells of the endocardial cushion undergoes endothelial-to-mesenchymal transition (EndMT) and provides the primary cell source for the development of AV valves [10]. Proliferation of the mesenchymal cells and matrix deposition extend the cushions into the cardiac lumen and form primordia of each distinct valve. This is followed by elongation and remodeling of the valve primordia, which leads to the gradual formation of valves by E15 [11]. The valves continue to grow and remodel throughout heart development and well after birth [12]–[14]. EndMT is a crucial process for proper formation of AV valves [15]. Transforming growth factor (TGFβ), bone morphogenetic protein (BMP)-2, and Snail1 have been shown to promote EndMT and valve formation [16]. We have shown that NOS3 is important for embryonic heart development [17]. Deficiency in NOS3 leads to congenital septal defect, bicuspid aortic valves and coronary artery malformation [18]–[20]. In addition, Notch-dependent NOS3 activation has been shown to promote EndMT in the developing AV canal from E9 to E11.5 via activation of the PI3-kinase/Akt pathway [21]. The temporal expression pattern of NOS3 peaks during AV valve formation along with its role in EndMT, suggesting that NOS3 may participate in the development of the AV valves. However, the role of NOS3 in the formation and functioning of AV valves has not been studied. In the present study, we hypothesized that NOS3 promotes embryonic development of AV valves via EndMT. To test this hypothesis, morphological changes and functional competence of AV valves were studied in wild-type (NOS3^+/+^), NOS3^−/−^ and cardiac specific NOS3 overexpressing (NOS3^Tg^) mice. Expression of human NOS3, driven by the β-myosin heavy chain promoter, was detected only in the embryonic heart of NOS3^Tg^ mice. The NOS3^Tg^ mice were then crossed with NOS3^−/−^ to create the NOS3^Tg^;NOS3^−/−^ mouse, an animal that lacks NOS3 in all organs except the heart during embryogenesis. Furthermore, the role of NOS3 in endocardial EndMT and signaling pathway critical to AV valve development was also examined. Our study demonstrated that NOS3 promotes endocardial EndMT and embryonic AV valve development.

## Results

### Deficiency in NOS3 Impairs AV Valve Formation

**Figure 1:**
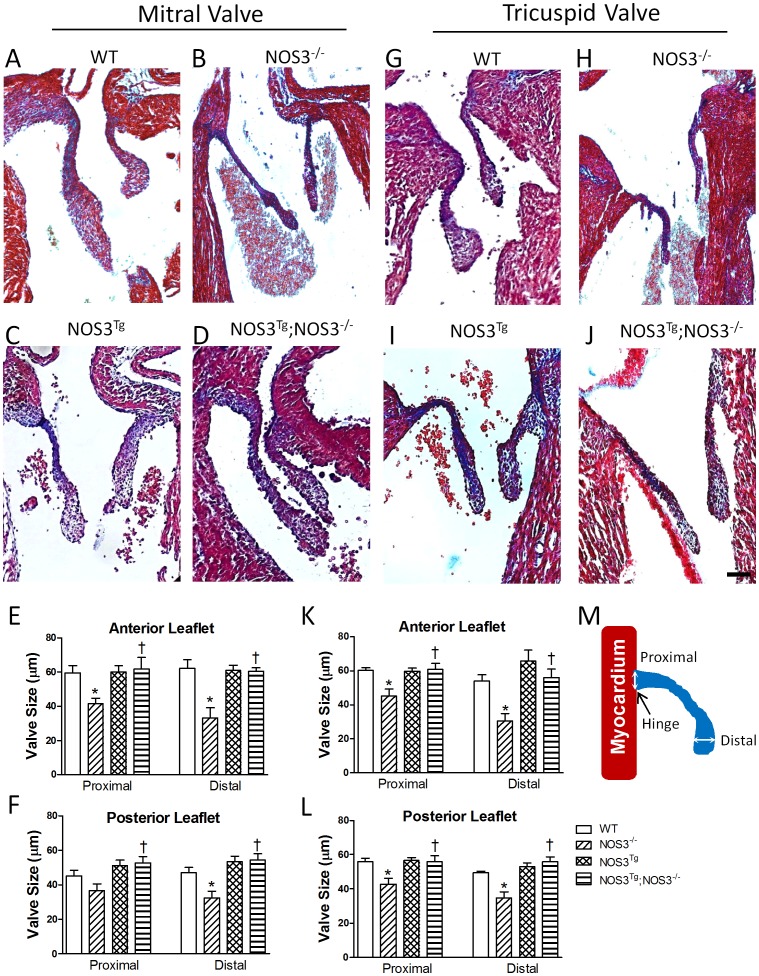
Malformation of mitral and tricuspid valves in NOS3^−/−^ mice, which are rescued by cardiomyocyte-specific NOS3 overexpression (NOS3^Tg^;NOS3^−/−^). Representative tissue sections with Masson’s trichrome staining of mitral (**A–D**) and tricuspid (**G–J**) valves in WT, NOS3^−/−^, NOS3^Tg^ and NOS3^Tg^;NOS3^−/−^ mice at P0. (**E** and **F**) Quantification of mitral valve size. (**K** and **L**) Quantification of tricuspid valve size. Anterior and posterior leaflets represent the valve leaflet closer to the septum and ventricular free wall, respectively. (**M**) Valve size was measured at the proximal (hinge) and distal aspects of the leaflets. White arrows represent proximal and distal measurements. Black arrow points to the hinge of the valve. Scale bar = 60 µm. Data are mean ± SEM from 5–8 mice per group. *P<0.05 vs. WT (NOS3^+/+^), ^†^ *P*<0.05 vs. NOS3^−/−^ mice.

Trichrome staining of mitral and tricuspid valves in NOS3^+/+^, NOS3^−/−^, NOS3^Tg^ and NOS3^Tg^;NOS3^−/−^ mice at P0 is shown in Figure 1A–D, G–J. In NOS3^−/−^ hearts, AV valves were much smaller than those of other genotypes. The size of proximal (hinge) and distal aspects of the valve leaflets was measured as illustrated in Figure 1M. Quantitative analysis of mitral and tricuspid valves show that the size and length of the proximal and distal leaflets were significantly smaller in the NOS3^−/−^ compared with NOS3^+/+^ hearts (*P*<0.05, Figure 1E–F, K–L, S1F, S2F). Cardiomyocyte-specific NOS3 overexpression (NOS3^Tg^;NOS3^−/−^) completely rescued these defects in the NOS3^−/−^ mice (*P*<0.05, Figure 1E–F, 1K–L, S1F, S2F). To investigate whether the valvular defect in the NOS3^−/−^ animals is due to premature maturation, cell density in the valves was measured [12]–[14]. Our data showed that there was no significant difference in cell density in mitral and tricuspid valves among all groups at P0 (Figure S1G and S2G). Furthermore, the decrease in valve length was evident as early as E15.5 in both mitral and tricuspid valves (Figure S3A–B). These data supports a developmental defect rather than premature maturation of the AV valves in NOS3^−/−^ mice.

The volume of the mitral and tricuspid valves was determined through 3D reconstructions (Figure S1A–E and S2A–E). The normal mitral valve of NOS3^+/+^ mice had 2 distinct leaflets. The overall volume of the mitral valve was significantly smaller in NOS3^−/−^ compared with NOS3^+/+^ mice, which was restored by cardiomyocyte-specific NOS3 overexpression in the NOS3^Tg^;NOS3^−/−^ mice (*P*<0.01, Figure S1A–E). The tricuspid valve of NOS3^+/+^ mice had 3 distinct leaflets. Similar to the findings in the mitral valve, the overall volume of tricuspid valve was significantly decreased in NOS3^−/−^ compared with NOS3^+/+^ mice, which was completely rescued by cardiomyocyte-specific NOS3 overexpression in the NOS3^Tg^;NOS3^−/−^ mice (*P*<0.01, Figure S2A–E).

### Regurgitation of AV Valves in NOS3^−/−^ Mice

**Figure 2:**
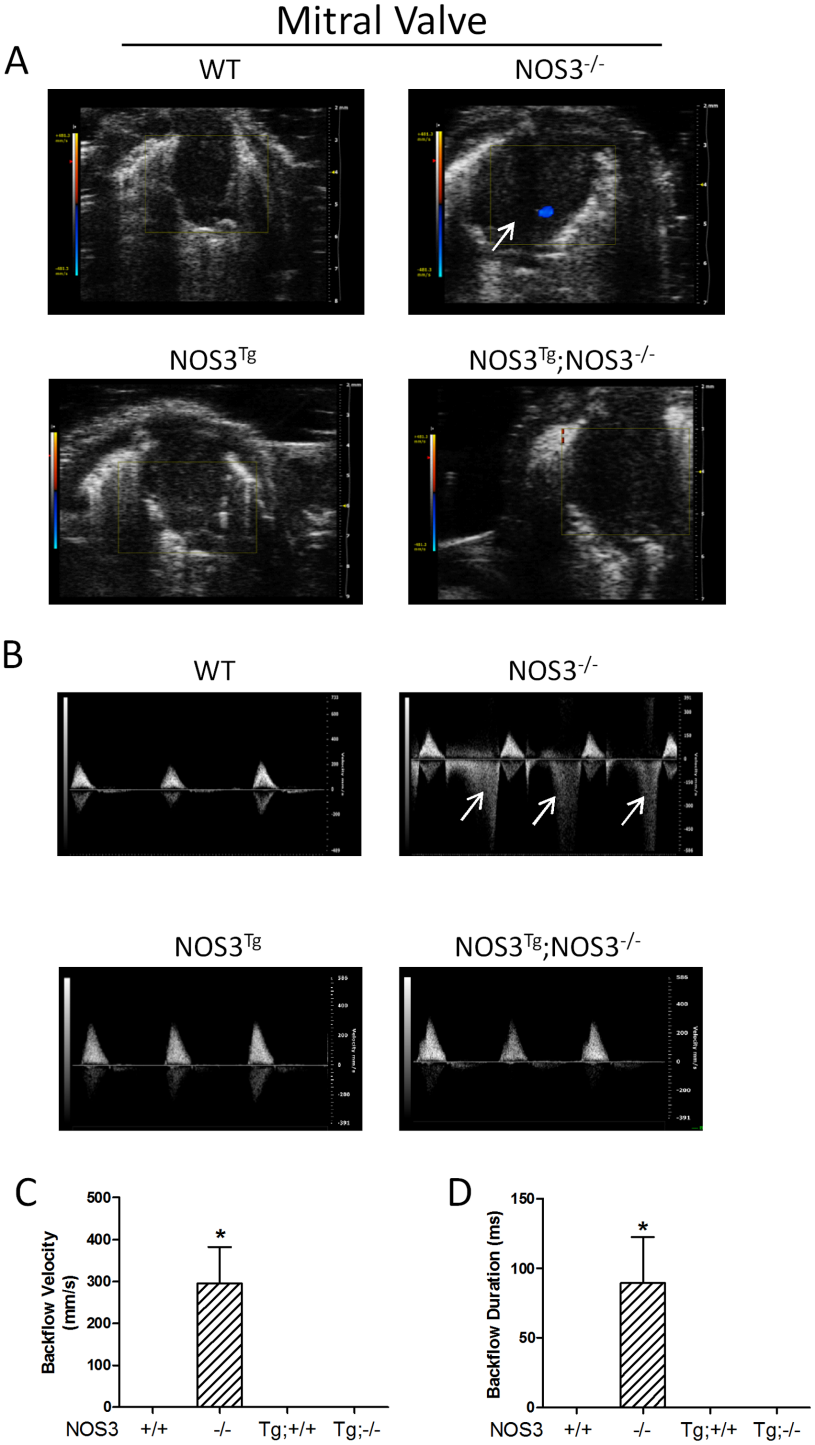
Regurgitation of mitral valve in NOS3^−/−^ mice at P0. Backflow of mitral valves was determined by color (**A**) and pulsed-wave (**B**) Doppler echocardiography. Backflow during systole is indicated by arrows. WT mice had no backflow in mitral valves. However, NOS3^−/−^ mice showed significant mitral valve backflow. (**C–D**) Quantification of mitral valve regurgitation. Significant regurgitation was observed in NOS3^−/−^ mice, which were rescued by cardiomyocyte-specific NOS3 overexpression. Data are mean ± SEM from 6–7 mice per group. *P<0.01 vs. all other groups. +/+, WT; −/−, knockout; Tg, transgenic. Tg;−/− and Tg;+/+ indicate NOS^Tg^;NOS3^−/−^ and NOS3^Tg^, respectively.

**Figure 3:**
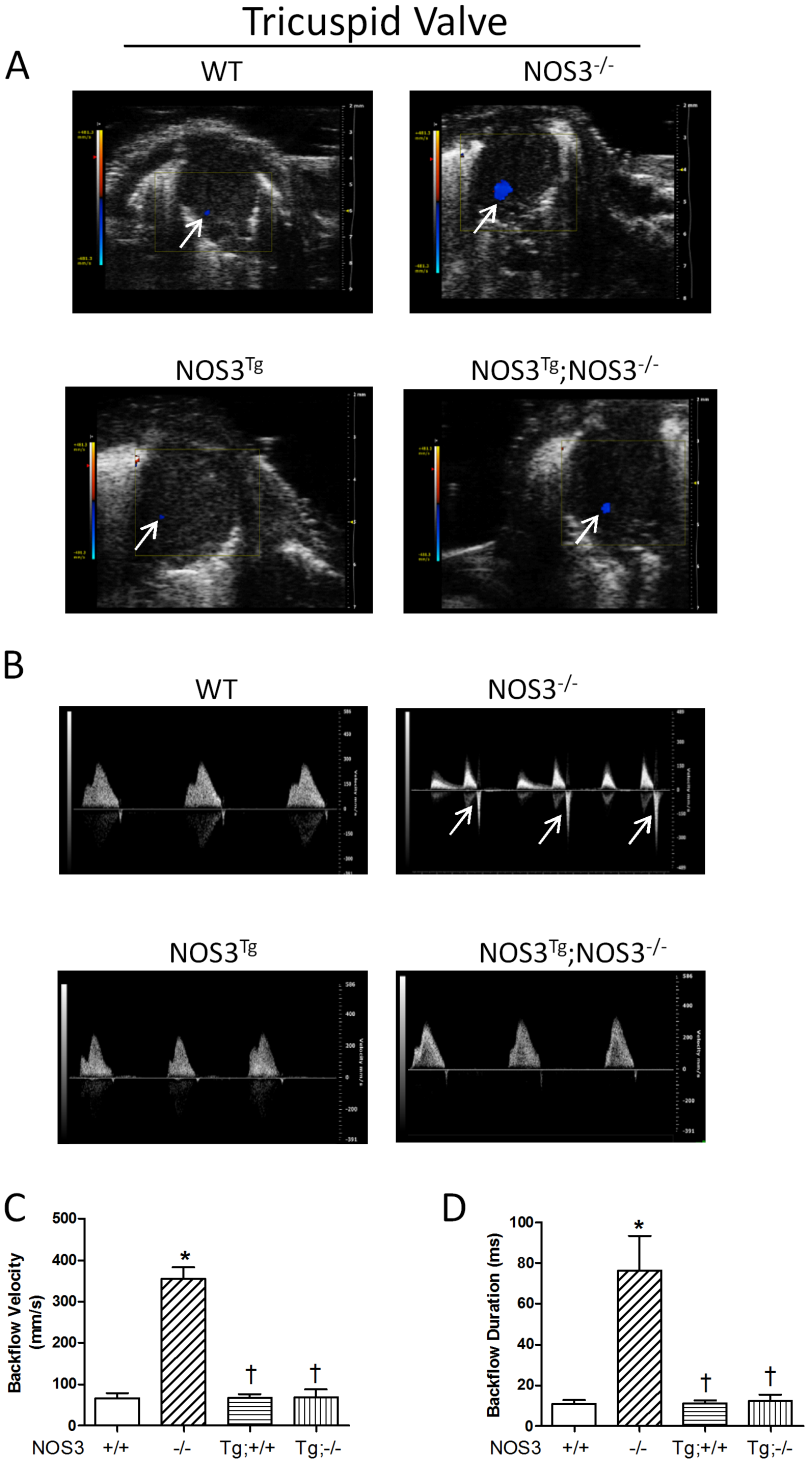
Regurgitation of tricuspid valve in NOS3^−/−^ mice at P0. Backflow of tricuspid valves was determined by color (**A**) and pulsed-wave (**B**) Doppler echocardiography. Backflow during systole is indicated by arrows. WT mice had minor backflow in tricuspid valves. However, NOS3^−/−^ mice showed marked tricuspid valve backflow. (**C–D**) Quantification of tricuspid valve regurgitation. Significant regurgitation was observed in NOS3^−/−^ mice, which were rescued by cardiomyocyte-specific NOS3 overexpression. Data are mean ± SEM from 6–7 mice per group. *P<0.01 vs. WT (NOS3^+/+^), ^†^ *P*<0.01 vs. NOS3^−/−^. +/+, WT; −/−, knockout; Tg, transgenic. Tg;−/− and Tg;+/+ indicate NOS^Tg^;NOS3^−/−^ and NOS3^Tg^, respectively.

In order to investigate the functional significance of AV malformation in NOS3^−/−^ mice, echocardiographic imaging analysis was performed on NOS3^+/+^, NOS3^−/−^, NOS3^Tg^ and NOS3^Tg^;NOS3^−/−^ mice at P0 live animals. Color flow Doppler recordings showed backflow of mitral (Figure 2A) and tricuspid (Figure 3A) valves in NOS3^−/−^ mice. Using pulsed-wave Doppler, the backflow velocity and duration were quantified. The velocity and duration of backflow in mitral (Figure 2B–D) and tricuspid valves (Figure 3B–D) were significantly increased in NOS3^−/−^ compared with NOS3^+/+^ mice (*P*<0.05). Cardiomyocyte-specific NOS3 overexpression completely rescued these abnormalities in the NOS3^−/−^ animals (Figure 2A–D, 3A–D).

**Figure 4:**
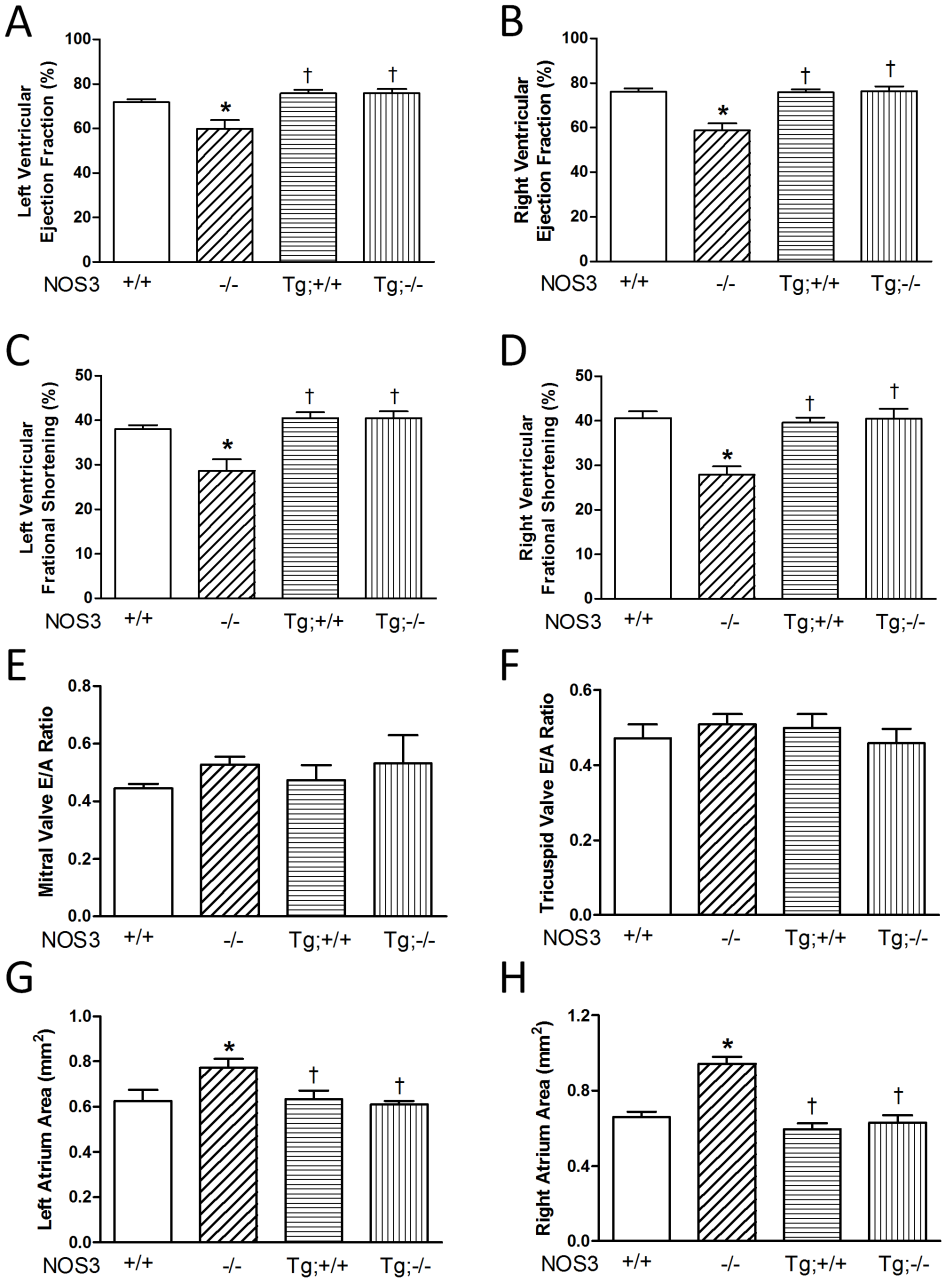
Cardiac function determined using echocardiography at P0. (**A–D**) Both left and right ventricular ejection fraction and fractional shortening were significantly decreased in the NOS3^−/−^ mice compared with WT controls, which were rescued by cardiomyocyte-specific NOS3 overexpression. (**E–F**) E/A ratio showed no significant difference between all groups. (**G–H**) Left and right atrial size was significantly increased in NOS3^−/−^ mice compared with WT controls, which was rescued by cardiomyocyte-specific NOS3 overexpression. Data are mean ± SEM from 6–7 mice per group. *P<0.05 vs. WT (NOS3^+/+^), ^†^ *P*<0.05 vs. NOS3^−/−^. +/+, WT; −/−, knockout; Tg, transgenic. Tg;−/− and Tg;+/+ indicate NOS^Tg^;NOS3^−/−^ and NOS3^Tg^, respectively.

To assess cardiac function, ejection fraction and fractional shortening were determined. Our data showed that ejection fraction and fractional shortening of both left and right ventricles were significantly decreased in NOS3^−/−^ compared with NOS3^+/+^ mice (*P*<0.05, Figure 4A–D). Cardiomyocyte-specific NOS3 overexpression in NOS3^−/−^ mice restored ejection fraction and fractional shortening in both left and right ventricles to levels comparable to the NOS3^+/+^ mice (Figure 4A–D). Additionally, we assessed the E/A ratio of the AV valves, which represents diastolic function or the ability of the blood to flow from the atria to the ventricles. E represents the passive filling of blood from atria to ventricles, whereas A represents the active contraction of the atria that pumps blood to the ventricles. Our results showed that no significant difference was observed between all groups in E/A ratios or the ability of the blood to flow from the atria to the ventricles (Figure 4E–F). Lastly, as a consequence of AV valve regurgitation, the size of atria would increase due to the backward flow from the ventricles. As expected, the size of both left and right atrium was significantly increased in the NOS3^−/−^ compared with NOS3^+/+^ mice, which was restored in NOS3^Tg^;NOS3^−/−^ mice at P0 (*P*<0.05, Figure 4G–H).

### Total Mesenchymal and Snail1^+^ Cells are Decreased in the Endocardial Cushion of NOS3^−/−^ Mice

**Figure 5:**
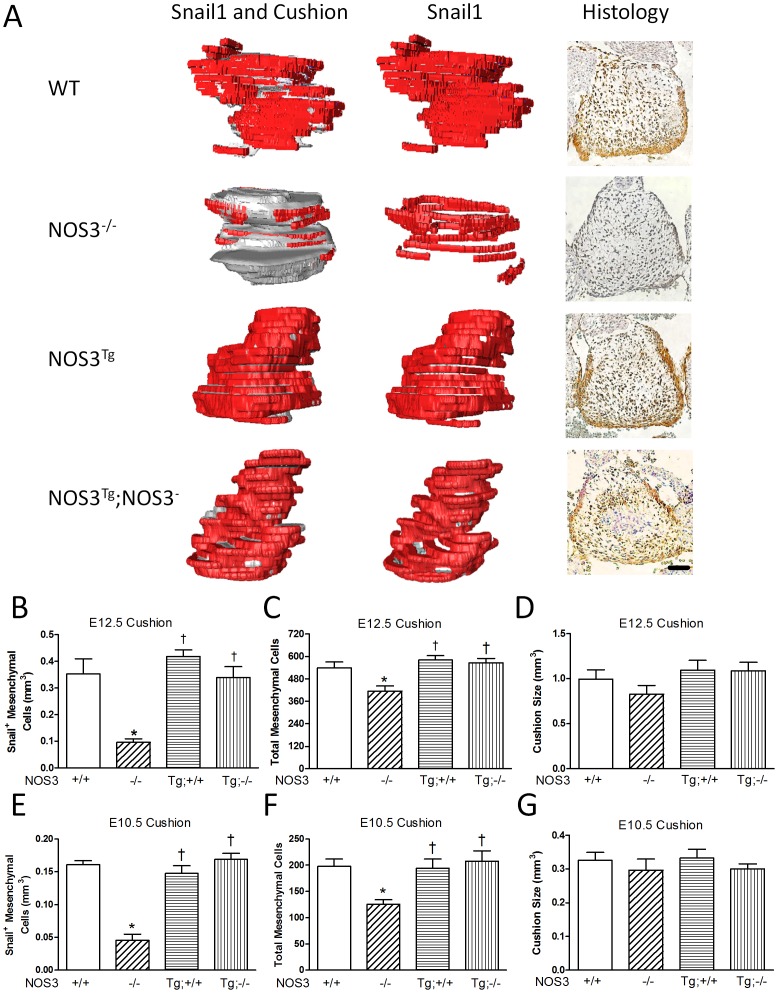
EndMT is impaired in the AV cushion of NOS3^−/−^ hearts at E12.5. (**A**) 3D reconstructions (Left and middle columns, Red for Snail1^+^ cells; Grey for cushion) and representative Snail1 immunostaining (Right column, Brown staining indicates Snail1^+^ cells). Scale bar = 50 µm. (**B**) Quantification of Snail^+^ mesenchymal cell volume from 3D reconstructed in E12.5 AV cushions. (**C**) Total number of mesenchymal cells (Snail positive and negative cells) in E12.5 cushions was quantified. (**D**) Quantification of endocardial cushion size at E12.5 from 3D reconstructions. (**E**) Quantification of Snail1^+^ endocardial cushion mesenchymal cells at E10.5 from 3D reconstructions. (**F**) Quantification of total endocardial cushion mesenchymal cells at E10.5. (**G**) Quantification of endocardial cushion size at E10.5 from 3D reconstructions. N = 3–4 mice per group. *P<0.05 vs. WT(NOS3^+/+^) or WT control. ^†^ *P*<0.05 vs. NOS3^−/−^.

Snail1 is a zinc finger transcriptional repressor, which inhibits adhesion molecule E-cadherin expression and induces EndMT during embryonic development [22], [23]. Therefore, Snail1 is an excellent marker of mesenchymal cells in the AV cushion. To further elucidate the role of NOS3 in early AV valve formation, immunostaining of Snail1 was performed and Snail1^+^ mesenchymal cells were quantified at E10.5 and E12.5. Our data showed that while there were no significant changes in the overall size and volume of the AV endocardial cushion at E10.5 and E12.5 (Figure 5D, G), both total mesenchymal and Snail1 positive cells (brown staining in histology) in the endocardial cushion were significantly decreased in NOS3^−/−^ compared with NOS3^+/+^ hearts (*P*<0.05, Figure 5A–C, E, F). Three-dimensional reconstruction of Snail1 expression (labeled in red) in the NOS3^+/+^ mice showed Snail1 completely covered the outer part of the endocardial cushion, which represents cells that are actively undergoing EndMT (Figure 5A). However, in the NOS3^−/−^ mice, only limited parts of the endocardial cushion were Snail1 positive, indicating the EndMT process is impaired in the NOS3^−/−^ mice. The number of Snail1 positive and total mesenchymal cells was completely restored by cardiac specific NOS3 overexpression (*P*<0.05, Figure 5A–C, E, F).

### cGMP Signaling is Decreased in NOS3^−/−^ Hearts

**Figure 6:**
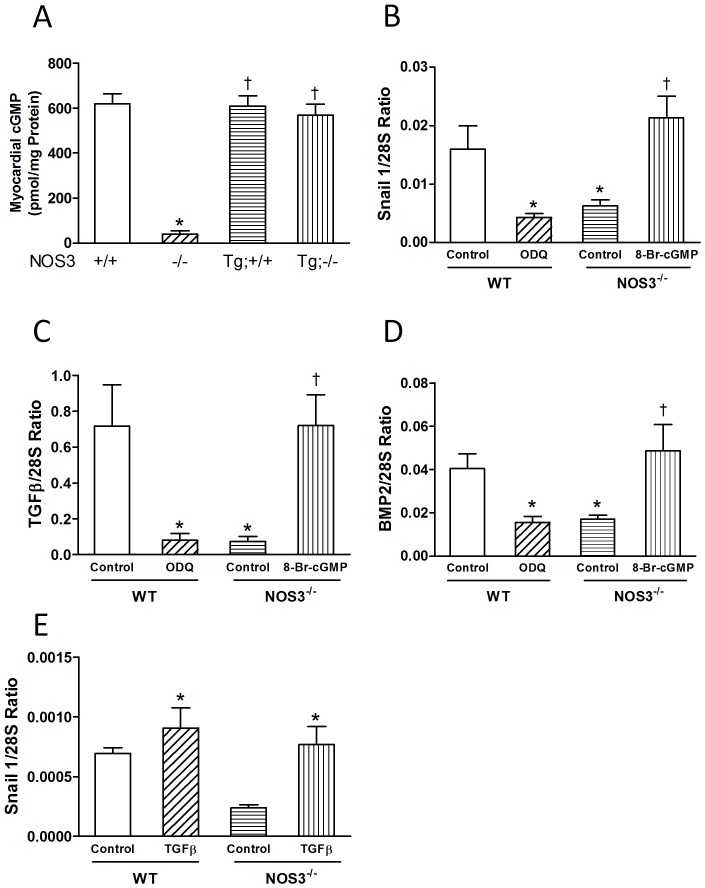
cGMP regulates expression of Snail1, TGFβ and BMP2 in E12.5 hearts. (**A**) cGMP levels in P0 hearts. (**B–D**) E12.5 *ex vivo* heart cultures. Cultured WT and NOS3^−/−^ hearts were treated with ODQ (100 µM), a soluble guanylate cyclase inhibitor, or 8-Br-cGMP (2 mM), a cGMP analog for 6 hrs. Snail1, TGFβ and BMP2 mRNA levels were determined by real-time RT-PCR. (**E**) E12.5 *ex vivo* heart cultures from WT and NOS3^−/−^ mice were treated with recombinant TGFβ protein (10 ng/ml). TGFβ mRNA levels were determined by real-time RT-PCR. N = 6 hearts per group for C–G. *P<0.05 vs. WT(NOS3^+/+^) or WT control. ^†^ *P*<0.05 vs. NOS3^−/−^ or NOS3^−/−^ control.

cGMP, a downstream signaling molecule of NOS3, was significantly decreased in the NOS3^−/−^ compared with NOS3^+/+^ hearts at P0 (*P*<0.05, Figure 6A). Cardiac specific NOS3 overexpression in NOS3^−/−^ mice increased myocardial cGMP to similar levels of NOS3^+/+^ mice (Figure 6A). To elucidate the precise role of NOS3 in regulating Snail1 expression, *ex vivo* E12.5 heart cultures were employed. The TGFβ and BMP family proteins are critical to AV valve development [15], [16]. Treatment with ODQ, a soluble guanylyl cyclase inhibitor significantly decreased TGFβ1, BMP2 and Snail1 mRNA expression in the NOS3^+/+^ hearts (*P*<0.05, Figure 6B–D). Conversely, NOS3^−/−^ hearts treated with 8-Bromo-cGMP, a cGMP analog showed a significant increase in TGFβ1, BMP2 and Snail1 mRNA levels (*P*<0.05, Figure 6B–D). These data suggest that NOS3 regulates the expression of TGFβ1, BMP2 and Snail1 through a cGMP-dependent pathway. To investigate if TGFβ regulates Snail1 signalling, recombinant TGFβ was used to treat NOS3^+/+^ and NOS3^−/−^ hearts. Our data showed that recombinant TGFβ protein treatment significantly increased Snail1 expression in NOS3^+/+^ and NOS3^−/−^ hearts (*P*<0.05, Figure 6E).

### EndMT is Impaired in NOS3^−/−^ Endocardial Cushions

**Figure 7:**
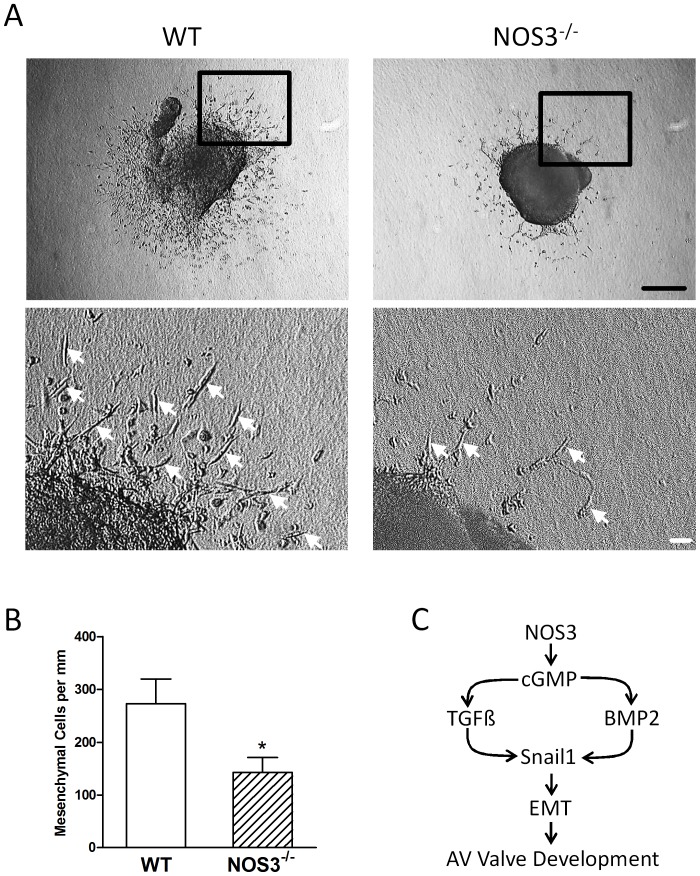
Atrioventricular (AV) endocardial cushion explant culture from E10.5 NOS3^−/−^ and WT mice. (**A**) Spindle shaped mesenchymal cells (arrows) were formed after 24 hours of culture. Lower panel is an enlargement of the boxed area. (**B**) The total number of mesenchymal cells per mm of explant cushion tissue was significantly decreased in NOS3^−/−^ compared to WT explants. Data are mean ± SEM from 6 explants per group. *P<0.05 vs. WT(NOS3^+/+^). Black scale bar = 100 µm, white scale bar = 10 µm. (**C**) Proposed signaling pathway by which NOS3 promotes endocardial EndMT and AV valve development.

To further confirm the role of NOS3 in endocardial EndMT, endocardial cushions from E10.5 embryos were dissected and cultured on collagen type I gel. Collagen type I gel was chosen to simulate the *in vivo* conditions of the cardiac jelly, which the mesenchymal cells migrate into. After 2 days of culture, cells that underwent EndMT exhibited spindle-like morphology and migrated from the endocardial cushion into the collagen gel. Results showed the total number of mesenchymal cells was significantly decreased in NOS3^−/−^ compared to NOS3^+/+^ explants (*P*<0.05, Figure 7A–B), suggesting NOS3 promotes endocardial EndMT.

## Discussion

The present study was carried out to examine the role of NOS3 in AV valve formation. We demonstrated for the first time that deficiency in NOS3 leads congenital AV valve defects. Additionally, lack of NOS3 also impairs endocardial EndMT. Furthermore, we showed that cardiomyocyte-specific NOS3 overexpression mice completely rescued these defects in NOS3^−/−^ mice. In order to assess the functional significance of these defects, echocardiography was carried out on P0 live animals. Our data showed severe regurgitation of both mitral and tricuspid valves in the NOS3^−/−^ mice. This phenotype was accompanied by reduced ejection fraction and fractional shortening and increased atrial size. Moreover, cardiomyocyte-specific NOS3 overexpression completely rescued these phenotypes. Our study suggests that NOS3 promotes endocardial EndMT and embryonic development of AV valves (Figure 7C).

The main function of AV valves is to direct blood flow from atria to ventricles, and from ventricles to the pulmonary and systemic circulations. Clinically, AV valve regurgitation can present in various forms depending on etiology and severity [23]. In the case of acute AV valve regurgitation, heart failure and cardiogenic shock may occur leading to death as a result of backflow from the ventricles and volume overload [24], [25]. Severe AV valve regurgitation, cardiac dysfunction and increases in atrial size were observed in NOS3^−/−^ mice. These phenotypes highly resemble congenital AV valve insufficiency, a clinical condition with infants born with shortened or a complete lack of AV valves [26], [27]. Mitral valve insufficiency is often associated with other cardiac defects such as atrial septal defects (ASD), ventricular septal defects (VSD), aortic valve insufficiency and tricuspid valve insufficiency [28]. Tricuspid valve insufficiency, also known as tricuspid valve regurgitation, is also associated with congenital cardiac defects such as VSD and mitral valve regurgitation [26]. These accompanying cardiac defects are very similar to phenotypes seen in the NOS3^−/−^ mice providing an excellent animal model to study AV insufficiency [18], [19]. Clinically, AV valve insufficiency is a serious congenital heart disease with very limited options other than invasive open-chest surgery. The molecular mechanisms underlying this disease are still largely unknown. The present study on the role of NOS3 in AV valve formation provides a possible mechanistic explanation to AV valve insufficiency.

Snail1, a zinc finger transcriptional repressor, is critical to induction of EndMT during embryonic development [22]. Snail1 knockout mice have defects in mesoderm formation and die around E7.5 [29]. In addition, deletion of Snai1 specifically in the epiblast results in death by E9.5 due to multiple vascular defects and increased apoptosis [30]. To investigate the role of NOS3 in early AV valve formation, *in vivo* Snail1 expression and *ex vivo* endocardial EndMT were assessed. Our results showed that both total mesenchymal and Snail1 positive cells in the endocardial cushion were significantly decreased in NOS3^−/−^ compared with NOS3^+/+^ hearts at E10.5 and E12.5 (Figure 5). In addition, the total number of mesenchymal cells from E10.5 endocardial cushion cultures was significantly decreased in NOS3^−/−^ explants (Figure 7A, B). These data show a reduction of endocardial EndMT in the NOS3^−/−^ mice leading to malformation of mitral and tricuspid valves.

TGFβ is expressed at high levels in AV mesenchyme during cushion formation and critical to EndMT and AV valve development [31]. Blockade of TGFβ2 by neutralizing antibody inhibits EndMT in mouse heart explants and TGFβ2 knockout mice show valvular defects [32], [33]. BMP2 is also important for AV cushion EndMT. Nkx2.5 mediated deletion of BMP2 reduced AV cushion formation and cellularity [34]. A classic pathway by which NO mediates its biological function is through cGMP-dependent protein kinase G (PKG) signaling [35]. In this regard, NO has been shown to upregulate TGFβ1 and BMP2 via PKG dependent pathway [36]–[38]. In order to study the molecular mechanism by which NOS3 promotes endocardial EndMT and AV valve development, *ex vivo* E12.5 heart cultures were carried out to investigate the role of NO/cGMP signaling in the expression of TGFβ1, BMP2 and Snail1. We showed that inhibition of guanylate cyclase decreased TGFβ1, BMP2 and Snail1 expression in the NOS3^+/+^ hearts while treatment with 8-Bromo-cGMP increased TGFβ, BMP2 and Snail1 mRNA levels in the NOS3^−/−^ hearts. These findings indicate that NOS3 acts as an upstream regulator of TGFβ1, BMP2 and Snail1 through a cGMP-dependent pathway. Notably, TGFβ1 and BMP are able to induce Snail1expression [15], [16]. To this end, we showed that recombinant TGFβ treatment significantly increased Snail1 expression in NOS3^+/+^ and NOS3^−/−^ hearts. Taken together, our data suggest that cGMP production from NOS3 upregulates TGFβ and BMP2, and promotes Snail1 expression and EndMT, leading to normal AV valve development (Figure 7C).

In summary, endocardial EndMT, an important process required for valvulogenesis is impaired in NOS3^−/−^ mice at E10.5 and E12.5, leading to malformation, insufficiency and regurgitation of AV valves at P0. We anticipate that these new insights into the mechanisms of AV valve development may lead to therapeutic strategies in the prevention and possibly treatment of AV valve insufficiency.

## Materials and Methods

### Animals

Breeding pairs of NOS3^−/−^ (stock No. 002684) and wild-type C57BL/6 (NOS3^+/+^) mice were purchased from Jackson Laboratory (Bar Harbor, Maine). A breeding program was carried out to produce neonates. Genotyping of NOS3^−/−^ and NOS3^+/+^ mice was performed by a polymerase chain reaction (PCR) method using genomic DNA prepared from tail biopsies. A timed breeding program was carried out. Vaginal plugging was monitored in the morning of each day and when plugged, was considered 0.5 days into pregnancy. Embryonic hearts were collected at E10.5, E12.5, and postnatal day 0. The investigation conforms to the *Guide for the Care and Use of Laboratory Animals* published by the National Institutes of Health (NIH Publication #85-23, revised 1996) and the experimental protocols were approved by Animal Use Subcommittee at Western University.

### Generation of Human NOS3 Transgenic Mice

A new line of cardiac-specific mice overexpressing human isoforms of NOS3 (NOS3^Tg^) was generated [20]. Briefly, human NOS3 complementary DNA was inserted into the β-myosin heavy chain promoter expression vector [39] to permit the expression of human NOS3 only during embryonic development and specifically in the heart. Our previous studies have shown that the transgene is specifically expressed in the heart during embryonic development [20]. Genotypes were identified by PCR using genomic DNA from tail biopsies.

### General Tissue Processing

Neonatal NOS3^+/+^, NOS3^−/−^, NOS3^Tg^/NOS3^−/−^ and NOS3^Tg^ mice were used for analysis. Hearts were isolated and fixed in 4% paraformaldehyde, dehydrated, and embedded in paraffin. Five micro-meter transverse serial sections were mounted onto albumin/glycerin coated glass slides.

### Immunohistochemistry and Immunofluorescence

Established protocols were used with minor modifications [18], [20], [40]. Briefly, after deparaffination and rehydration of the slides, microwave antigen retrieval was applied by heating them for 10 min at 98°C in a citric acid buffer (0.01 M in aqua-dest, pH 6.0). Inhibition of endogenous peroxidase was performed with a solution of 0.3% H_2_O_2_ in phosphate buffered saline (PBS) for 30 min. The slides were incubated overnight with either 1∶500 anti-Snail1 (Abcam, USA). Next, the goat anti-rabbit secondary antibody conjugated with biotin (1∶200, Vector Laboratories, USA) was added for 60 min in PBS. Subsequently, slides were incubated with ABC reagent (Vector Laboratories, USA) for 30 min. For visualization, the slides were incubated with 400 µg/ml 3–3′di-aminobenzidin tetrahydrochloride (Sigma-Aldrich, USA) and 100 µL of 30% H_2_O_2_ dissolved in PBS for 10–15 min. Counterstaining was performed with 0.1% hematoxylin. The size and length of AV valves were measured at the proximal (hinge) and distal aspects of the leaflets as described previously [41].

### Three-Dimensional Reconstructions and Histological Analysis

Three-dimensional (3D) reconstructions of the heart and AV valves were performed using Masson’s trichrome stained serial sections of P1 hearts through the AMIRA® software (Template Graphics Software, USA) as previously described [20]. AV valve volumes were determined using the AMIRA® program.

### Heart Function Measurements

Left ventricular (LV) and right ventricular ejection fraction and fractional shortening were measured using the Vevo 2100 ultrasound imaging system (Visual Sonics, Canada) [20]. Briefly, 2-dimensional images of the heart were obtained in short-axis view using a dynamically focused 40 MHz probe. The M-mode cursor was positioned perpendicular to the LV anterior and posterior walls. The LV internal end-diastolic dimension (LVIDd) and LV internal systolic dimension (LVIDs) were measured from M-mode recordings. LV ejection fraction was calculated as: EF (%) = [(LVIDd)^3^−(LVIDs)^3^]/(LVIDd)^3^×100. Fractional shortening was calculated as: FS (%) = (LVIDd−LVIDs)/LVIDd×100. The M-mode measurements of the left ventricular ejection fraction and fractional shortening were averaged from 3 cycles. Both left and right atrial areas were traced and calculated under the apical 4-chamber view in B-mode. AV valve E/A ratio, regurgitation and flow pattern were measured through both color flow Doppler recordings and pulsed-wave Doppler echocardiograms [42].

### *Ex vivo* Heart Cultures

E12.5 NOS3^+/+^ and NOS3^−/−^ hearts were explanted and cultured in M199 medium (Sigma-Aldrich, USA) supplemented with 10% FBS as previous described [20]. Hearts were stabilized for 2 hours prior to any treatment. NOS3^+/+^ and NOS3^−/−^ hearts were beating throughout the entirety of the experiment. NOS3^+/+^ and NOS3^−/−^ hearts were treated with 100 µM 1H-[1], [2], [4]oxadiazolo[4,3,-a]quinoxalin-1-one (ODQ) (Sigma-Aldrich, USA) and 2 mM 8-Bromo-cGMP (Sigma-Aldrich, USA), respectively for 6 hours. Some NOS3^+/+^ and NOS3^−/−^ hearts were treated with recombinant TGFβ protein (10 ng/ml) for 18 hours. Hearts were then collected and mRNA levels of Snail1, TGFβ and BMP2 were analyzed by real time RT-PCR.

### Real-time RT-PCR

Standard protocol was used as previous described [7], [18], [20], [40]. Briefly, total RNA was isolated from cultured fetal hearts with a RNA isolation kit (Qiagen, Canada). cDNA was synthesized using M-MLV (Invitrogen, Canada) reverse transcriptase and random primers (Invitrogen, Canada). Real-time PCR was conducted using SYBR Green PCR Master Mix as per manufacturer’s instructions (Eurogentec, USA). The oligonucleotide primers used in this study are summarized in Table S1. Samples were amplified for 35 cycles using Eppendorf Real-Time PCR machine and analyzed using cycle threshold (Ct) analysis. The expression levels of Snail1, BMP2 and TGFβ in relation to 28S rRNA were determined using a comparative Ct method [40].

### Measurement of cGMP

The cGMP levels in the heart were measured using an ELISA kit according to the manufacturer’s instructions (ADI-900-014, Enzo Life Sciences, USA) [43]. Neonatal NOS3^+/+^, NOS3^−/−^, NOS3^Tg^/NOS3^−/−^ and NOS3^Tg^ hearts were used for analysis. Briefly, 10 µg of protein lysates from the isolated hearts and standards were added to the cGMP conjugated to alkaline phosphatase and an anti-cGMP antibody, and incubated for 2 hours at room temperature. This incubation allows the antibody to bind the cGMP in the sample or conjugate in a competitive manner. After 3 washes, p-nitrophenyl phosphate (pNpp) substrates were added and the plated and incubated for 1 hour at room temperature. This incubation allows the catalysis of pNpp substrate by the alkaline phosphatase on the cGMP conjugate. The reaction was then stopped by tri-sodium phosphate solution and the optical density was read at 405 nm. The amount of signal is indirectly proportional to the amount of cGMP in the sample.

### Endocardial Cushion Explant Culture

Endocardial cushion explants were cultured accordingly to methods previously described with minor modifications [32]. Briefly, E10.5 NOS3^+/+^ and NOS3^−/−^ hearts were planted on a 35 mm petri dish coated with 1 mg/ml collagen type I gel overnight for attachment. After attachment, the hearts were treated with 100 µl of M199 supplemented with 10% FBS and allowed 2 days for EndMT to occur. After 2 days, these hearts were imaged under phase contrast microscopy (Observer D1, Zeiss, Germany). The number of spindle-like mesenchymal cells was counted.

### Statistical Analysis

Data are presented as mean ± SEM. Unpaired Student’s *t* test was used for 2 group comparisons. One way ANOVA followed by Student-Newman-Keuls test was performed for multi-group comparisons. *P*<0.05 was considered statistically significant.

## Supporting Information

Figure S1 Three-dimensional (3D) reconstructions of mitral valve of WT, NOS3^−/−^, NOS3^Tg^ and NOS3^Tg^;NOS3^−/−^ mice at P0 (**A–D**). 3D reconstructions were made by putting together images taken from approximately 100 serial heart sections at 5 µm using Amira software. Valve locations in the heart are shown. Whole heart images are frontal views of the heart. Isolated valve images are viewed from the left atrium into the left ventricle. **E**. Quantitative analysis of mitral valve volume. Data are mean ± SEM from 5 mice per group. **F**. Quantification of mitral valve length in P0 hearts. **G**. Quantification of mitral valve cell density in P0 hearts. *P<0.01 vs. WT. ^†^ *P*<0.01 vs. NOS3^−/−^ mice. Tg;−/− and Tg;+/+ indicate NOS^Tg^;NOS3^−/−^ and NOS3^Tg^, respectively.
(TIF)

Figure S2 3D reconstructions of tricuspid valve of WT, NOS3^−/−^, NOS3^Tg^ and NOS3^Tg^;NOS3^−/−^ mice at P0 (**A–D**). 3D reconstructions were made by putting together images taken from approximately 100 serial heart sections at 5 µm using Amira software. Valve locations in the heart are shown. Whole heart images are frontal views of the heart. Isolated valve images are viewed from the right atrium into the right ventricle. **E**. Quantitative analysis of tricuspid valve volume. **F**. Quantification of tricuspid valve length in P0 hearts. **G**. Quantification of tricuspid valve cell density in P0 hearts. Data are mean ± SEM from 5 mice per group. *P<0.01 vs. WT. ^†^ *P*<0.01 vs. NOS3^−/−^ mice. Tg;−/− and Tg;+/+ indicate NOS^Tg^;NOS3^−/−^ and NOS3^Tg^, respectively.
(TIF)

Figure S3 Mitral and tricuspid valve length measurements at E15.5. **A**. Length of anterior and posterior leaflets of the mitral valve. **B**. Length of anterior and posterior leaflets of the tricuspid valve. Data are mean ± SEM from 5 mice per group. *P<0.05 vs. WT. ^†^ *P*<0.05 vs. NOS3^−/−^ mice. Tg;−/− and Tg;+/+ indicate NOS^Tg^;NOS3^−/−^ and NOS3^Tg^, respectively.
(TIF)

Table S1 **Gene name, accession number and primers used.**
(DOC)
