# Supplementary figures and images for: Nitric Oxide Synthase-3 Promotes Embryonic Development of Atrioventricular Valves
Source: PLoS One. 2013 Oct 29;8(10):e77611. doi: 10.1371/journal.pone.0077611 (PMC3812218; doi:10.1371/journal.pone.0077611)

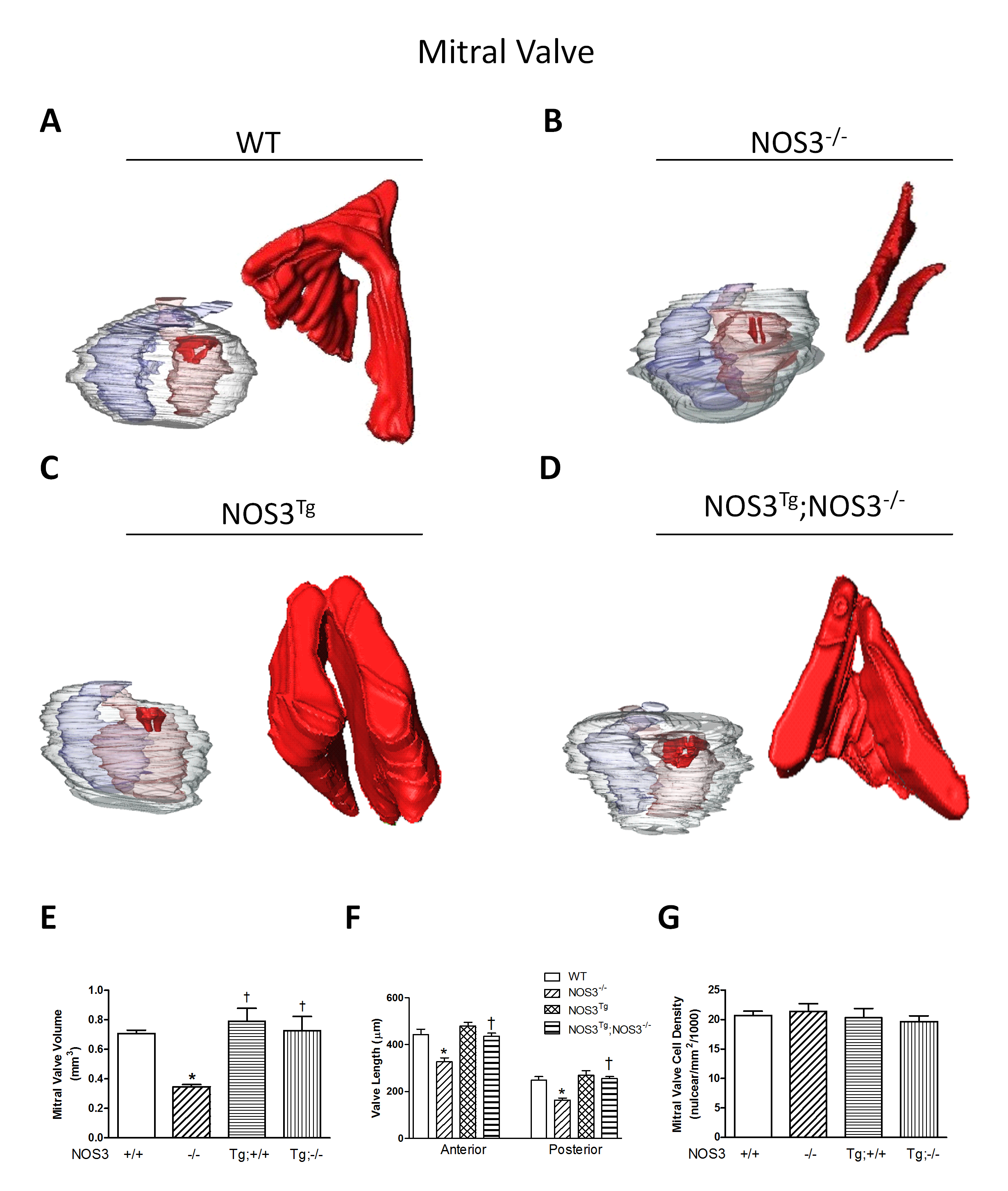

Supplement: Figure S1 — Three-dimensional (3D) reconstructions of mitral valve of WT, NOS3−/−, NOS3Tg and NOS3Tg;NOS3−/− mice at P0 (A–D). 3D reconstructions were made by putting together images taken from approximately 100 serial heart sections at 5 µm using Amira software. Valve locations in the heart are shown. Whole heart images are frontal views of the heart. Isolated valve images are viewed from the left atrium into the left ventricle. E. Quantitative analysis of mitral valve volume. Data are mean ± SEM from 5 mice per group. F. Quantification of mitral valve length in P0 hearts. G. Quantification of mitral valve cell density in P0 hearts. *P<0.01 vs. WT. † P<0.01 vs. NOS3−/− mice. Tg;−/− and Tg;+/+ indicate NOSTg;NOS3−/− and NOS3Tg, respectively. (TIF) [file pone.0077611.s001.tif]

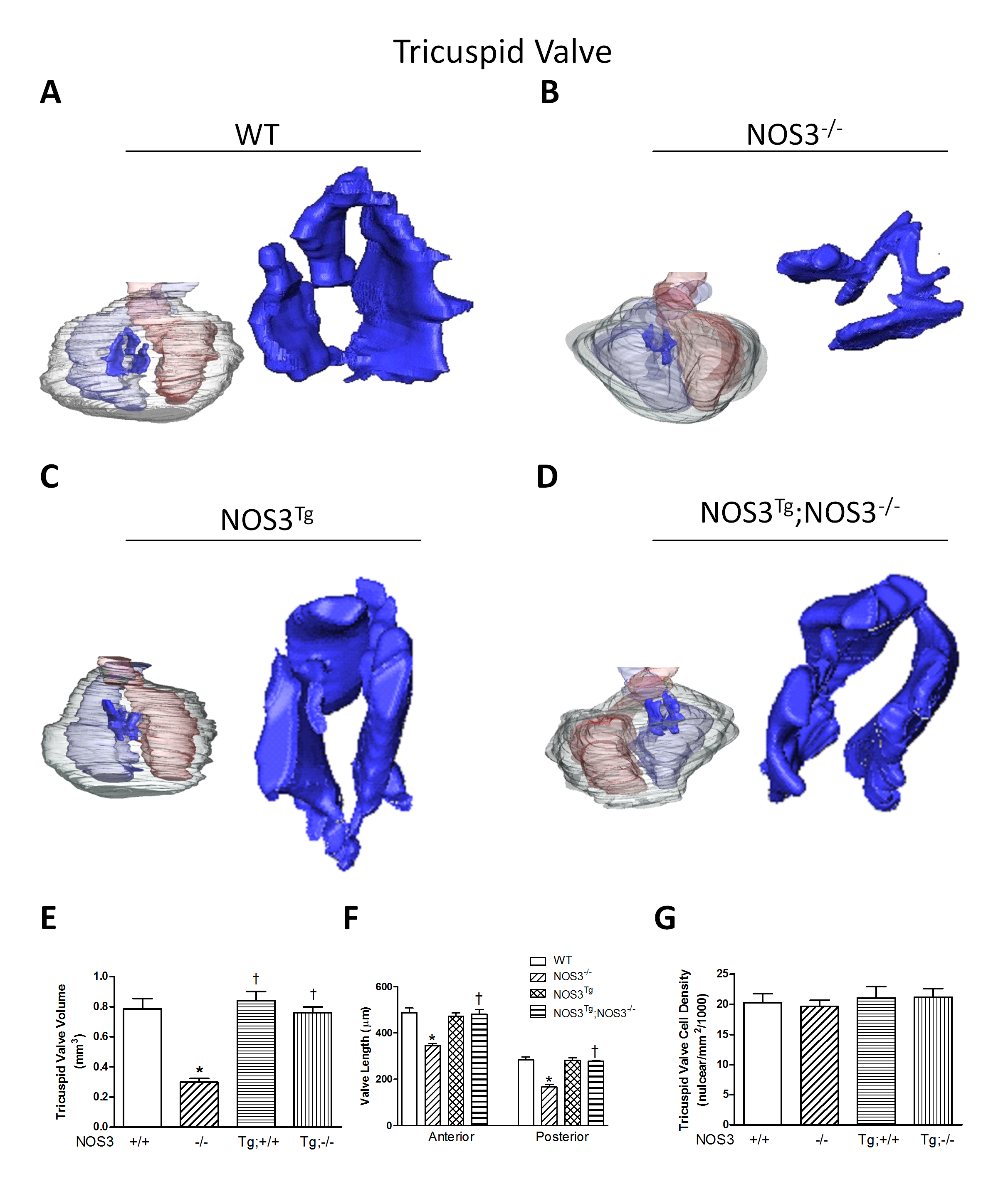

Supplement: Figure S2 — 3D reconstructions of tricuspid valve of WT, NOS3−/−, NOS3Tg and NOS3Tg;NOS3−/− mice at P0 (A–D). 3D reconstructions were made by putting together images taken from approximately 100 serial heart sections at 5 µm using Amira software. Valve locations in the heart are shown. Whole heart images are frontal views of the heart. Isolated valve images are viewed from the right atrium into the right ventricle. E. Quantitative analysis of tricuspid valve volume. F. Quantification of tricuspid valve length in P0 hearts. G. Quantification of tricuspid valve cell density in P0 hearts. Data are mean ± SEM from 5 mice per group. *P<0.01 vs. WT. † P<0.01 vs. NOS3−/− mice. Tg;−/− and Tg;+/+ indicate NOSTg;NOS3−/− and NOS3Tg, respectively. (TIF) [file pone.0077611.s002.tif]

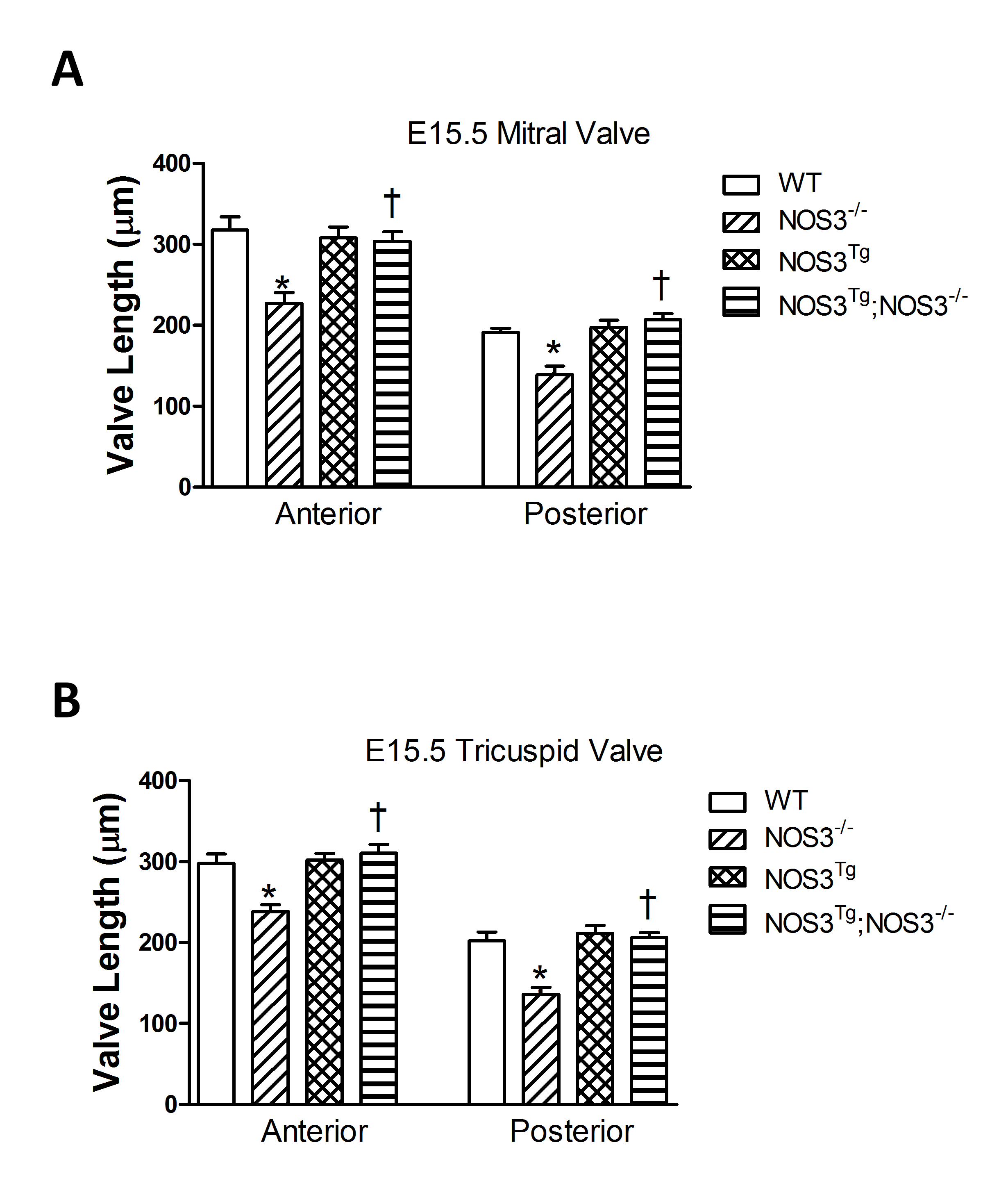

Supplement: Figure S3 — Mitral and tricuspid valve length measurements at E15.5. A. Length of anterior and posterior leaflets of the mitral valve. B. Length of anterior and posterior leaflets of the tricuspid valve. Data are mean ± SEM from 5 mice per group. *P<0.05 vs. WT. † P<0.05 vs. NOS3−/− mice. Tg;−/− and Tg;+/+ indicate NOSTg;NOS3−/− and NOS3Tg, respectively. (TIF) [file pone.0077611.s003.tif]
