# Supplementary material for: Nitric Oxide Synthase-3 Promotes Embryonic Development of Atrioventricular Valves
Source: PLoS One. 2013 Oct 29;8(10):e77611. doi: 10.1371/journal.pone.0077611 (PMC3812218; doi:10.1371/journal.pone.0077611)
Supplement: Table S1 — Gene name, accession number and primers used. (DOC) [file pone.0077611.s004.doc]

**Table S1. Gene name, accession number and primers used.**

| Gene | Accession # | Primer Sequence |
| --- | --- | --- |
| Snail1 | NM_011427.2 | Sense: 5’ CAC ACG CTG CCT TGT GTC T 3’  Antisense: 5’ GGT CAG CAA AAG CAC GGT T 3’ |
| BMP2 | NM_007553.2 | Sense: 5’ CAA ACA CAA ACA GCG GAA GC 3’  Antisense: 5’ CAG CAA GGG GAA AAG GAC AC 3’ |
| TGFβ | NM_011577.1 | Sense: 5’ CAC TGC TTC CCG AAT GTC TG 3’  Antisense: 5’ GCC CGA AGC GGA CTA CTA TG 3’ |
| 28S | NR_003279.1 | Sense: 5’ TTG AAA ATC CGG GGG AGA G 3’  Antisense: 5’ ACA TTG TTC CAA CAT GCC AG 3’ |
